# Screening young children for neurodevelopmental differences in sub-Saharan Africa: a scoping review

**DOI:** 10.1186/s12888-025-07279-0

**Published:** 2025-09-15

**Authors:** Ben Truter, Amy L. Slogrove, Elif Ilhan, Petra Conradie, Lucy Thompson, Christopher Gillberg, Eva Billstedt

**Affiliations:** 1 Gillberg Neuropsychiatry Centre, Sahlgrenska Academy, University of Gothenburg, Gothenburg, Sweden; 2 Ukwanda Centre for Rural Health, Department of Global Health, Faculty of Medicine & Health Sciences, Stellenbosch University, Stellenbosch, South Africa; 3 Department of Paediatrics & Child Health, Faculty of Medicine & Health Sciences, Stellenbosch University, Stellenbosch, South Africa; 4 The Neurodiversity Centre, Paarl, South Africa; 5 Institute of Applied Health Science, Centre for Health Science, University of Aberdeen, Inverness, UK

**Keywords:** Children Screening Neurodevelopment Africa Review

## Abstract

**Background:**

Neurodevelopmental disorders (NDDs) present in approximately 10% of children globally. Awareness of NDDs in sub-Saharan Africa is relatively low and little is known about prevalence. This scoping review aims to understand screening of NDDs in young children in sub-Saharan Africa, which NDDs are screened for, and who administers screening.

**Methods:**

The review was conducted in accordance with the PRISMA-ScR standards for scoping reviews. PubMed, Web of Science, SCOPUS and PsycInfo were searched according to a defined search strategy with relevant keywords. Studies published in English between 2012 and 2023, where children aged *≥* 2 to < 9 years years, residing in sub-Saharan Africa, were screened for a form of NDD, were included. Due to the flexible nature of a scoping review and its focus on under-explored topics, there were no restrictions on study design. Titles and abstracts were reviewed for selection independently by three researchers and full text articles independently by two researchers. Quality assessment was conducted using the Newcastle-Ottawa Scale.

**Results:**

After duplicate removal and title and abstract screening, 546 abstracts were retained for full text review. Twelve publications met the inclusion criteria. These were from South Africa (*n* = 6), Kenya (*n* = 3), Uganda (*n* = 2) and Malawi (*n* = 1). Only two explicitly screened for multiple NDDs. There was considerable heterogeneity in NDD conceptualisation, study design, screener design, populations of children screened, and persons performing the screening – limiting the possibility for valid comparative analysis. Quality assessment of methodologies yielded ratings ranging from “Very good” to “Unsatisfactory” (cross-sectional studies) and “Good” to “Fair” (cohort- and case-control studies).

**Conclusion:**

There are very few published studies on NDD screening in young children in sub-Saharan Africa. Further research examining simple, contextually appropriate screening for a wide range of NDDs amongst this group, is needed. This may inform future screening programmes, policy development and clinical practice throughout the region.

**Supplementary Information:**

The online version contains supplementary material available at 10.1186/s12888-025-07279-0.

## Introduction

The United Nations 2030 Agenda for Sustainable Development has shifted our focus from global child survival to ensuring that every child thrives and achieves their full developmental potential [1]. Healthcare research in sub-Saharan Africa (sSA) has traditionally focussed on communicable diseases like HIV and malaria, along with initiatives to reduce infant mortality [2–4]. Neurodevelopmental disorders, as defined by DSM-5, which we will further describe as neurodevelopmental differences (NDDs) may significantly hinder a child from reaching their potential but are an under-researched area in sSA. Few prevalence studies have been done, perhaps partly due to the lack of screening instruments validated in, and/or designed for the cultural contexts of, sSA. Large scale prevalence studies require reliable screening followed by detailed diagnostic assessment in those who screen positive. This scoping review explores current published research on screening for NDDs in young children in sSA.

NDDs may impact various aspects of a child’s functioning, including cognition, communication, social interaction, and motor skills [5–7]. Prominent among NDDs are attention-deficit/hyperactivity disorder (ADHD), specific learning disorders, autism, impediments in speech, language, and communication, and general learning difficulties [8–10]. The frequent co-occurrence of NDDs complicates the processes of assessment and diagnosis, as well as the selection of effective interventions, often rendering them intricate, protracted, and costly under the current intervention and support paradigm [11–13].

The acronym ESSENCE (Early Symptomatic Syndromes Eliciting Neurodevelopmental Clinical Examinations) was introduced by Prof Christopher Gillberg to underscore the interconnected, interwoven nature of the symptoms of various NDDs in early childhood [14]. Different NDDs will often exhibit shared symptoms and features during early development, complicating their differentiation based solely on observed behaviours [14–16]. A comprehensive, multi-disciplinary approach to challenges in the broader field of child health and development services, is needed [14, 15].

Increasing recognition of the co-occurrence of multiple NDDs [9] has led to recommendations that screening for all NDDs simultaneously is required to inform prevalence studies in low- and middle-income countries (LMIC) [8], to guide further research and policy development.

NDDs may persist across the lifespan and have intergenerational impacts [8, 17]. They may impose a substantial health and financial burden on families and society [18–20]. Early investment in the health, familial, social, and broader environmental contexts for children, has proved to be more cost-effective than addressing health and developmental issues later in life [21, 22].

Understanding the prevalence of NDDs among children is crucial for informing public health research and policy decisions [23–25]. From the few NDD prevalence studies in sub-Saharan Africa (sSA) observed rates have ranged widely between 2.9% and 18.7% [6, 26, 27]. Namazzi et al. [6] highlighted that inconsistencies in reported prevalence rates across the African continent are due to differing definitions of NDDs, variations in age of assessed children, and the use of various measurement tools. Context-specific socio-cultural- and language factors and differences may affect symptom presentation, understandings and responses to symptoms and signs of NDDs as well as help-seeking behaviour.

Screening and assessment for NDDs are especially relevant in environmentally deprived settings in sSA, where access to specialised healthcare may be limited [8, 27]. Children in LMICs, such as those in sSA, often confront a multitude of adverse conditions stemming from structural disadvantages [28, 29]. These include inadequate sanitation, poverty, overcrowded living arrangements, insufficient nutrition, limited access to psychosocial stimulation, and exposure to violence [22, 27], all of which add to the accumulated disadvantage experienced by children in these settings. Additional health factors, such as maternal mental health [30] and the adverse exposures experienced by children born to women living with HIV, must be considered in this package of early childhood exposures [31].

Early adversities have profound effects on children’s educational attainment [21] and may lead to delayed physical, social, emotional, and cognitive development - potentially contributing to NDDs among children in LMICs [27, 32, 33]. Numerous early adverse childhood events (ACEs) have been associated with neurodevelopmental challenges, including motor dysfunction, language and cognitive impairment, developmental delays, and social-behavioural issues [6, 34–36].

Screening is defined as a brief, easily administered test that does not result in formal diagnosis but can indicate elevated risk of a certain condition, whilst NDD assessment is a more comprehensive, clinical evaluation that is conducted after screening, to clarify diagnosis and guide intervention. Screening for NDDs may indicate whether a child is on the expected developmental track, allowing for further referral if concerns are identified [37]. Many, if not most, screening tools have been developed and validated in high income countries. These screening tools may not be cross-culturally applicable and may be inadequately translated when used in other settings, which could affect screening scores. Additionally, in sSA a considerable amount of public health care screening is performed by auxiliary health workers or persons who may possess little to no formal training in the conditions for which they are screening. There is thus a need to contribute to and develop further understanding of current screening processes, as well as possible factors that render screening processes and instruments feasible for implementation within sSA, as early NDD screening should form the first pathway to intervention for these children and their families.

The present review aims to establish the scope of existing literature on screening for NDDs in young children in sSA. Scoping reviews may be usefully employed in under-explored areas of study to identify and consider existing research.

### Objective

This review sought to examine existing research into the screening of children aged *≥* 2 to < 9 years in sSA and to answer the question: For what NDDs are children age ≥ 2 to < 9 years years in sSA being screened, what screening tools are being used, and who is administering the screens? A secondary objective was to examine factors that optimise or constrain the benefits of screening instruments used, or factors that facilitate an effective screening programme, if these are described in the studies found.

## Methods

The scoping review was conducted in accordance with the *Preferred Reporting Items for Systematic Reviews and Meta-analyses Extension for Scoping Review* (PRISMA-ScR) standards (Appendix A) [38]. PubMed, Web of Science, SCOPUS and PsycInfo were searched according to a defined search strategy, developed with input from a librarian, including key words such as “neurodevelopmental”, “screening”, “child” and the names of all sSA countries (full list reported in Appendix B).

### Inclusion criteria

We searched for studies that screened for NDDs as conceptualised in the ESSENCE framework [39]: ADHD with or without Oppositional Defiant Disorder/Conduct Disorder (ODD/CD); Language Disorder including antecedents of dyslexia; Developmental Coordination Disorder (DCD); Autism; Intellectual Disability/Intellectual Developmental Disorder (IDD); Borderline Intellectual Functioning; Tic disorders/Tourette syndrome; Obsessive Compulsive Disorder; Behavioural Phenotype Syndromes including Fetal Alcohol Spectrum Disorders; epilepsy syndromes; other neurological/neuromuscular disorders including Cerebral Palsy, Sturge-Weber Syndrome, Duchenne muscular dystrophy, myotonic dystrophy, Landau-Kleffner Syndrome; febrile seizures; HIV-associated Neurocognitive Disorder Paediatric Acute-onset Neuropsychiatric Syndrome (PANS); Paediatric autoimmune neuropsychiatric disorders associated with Streptococcus infections (PANDAS); and any global or domain specific developmental delay or cognitive/neurocognitive impairment.

Only studies published in English were included for reasons of feasibility and resource availability. Only studies published from January 2012 until December 2023 were included, to ensure current relevance. Studies had to include participants who were (i) *≥* 2 to < 9 years of age and who were potentially in either formal or informal preschool or care or early education settings, or in the first grade of school; and (ii) residing in sSA.

### Exclusion criteria

Studies making use of diagnostic instruments, tools or procedures only, without using screening tools, were excluded; as were studies of visual and/or auditory impairments only. Due to the flexible nature of a scoping review and the aim to scope literature in an under-explored topic there were no restrictions on study design, including qualitative and quantitative studies, or other research methodological considerations.

### Search strategy

The search strategy aimed to locate published studies. Unpublished studies or grey literature was not included. Scoping and systematic reviews were considered secondary sources and were therefore also excluded but were reviewed for relevance to our topic. An initial limited search of PubMed was undertaken to identify articles on the topic. The text words contained in the titles and abstracts of relevant articles, and the index terms used to describe the articles were then used to develop a full search strategy for PubMed, Web of Science, SCOPUS and PsycInfo. The search strategy underwent appropriate adjustments for each included database, incorporating specific keywords, index terms, and database specifications identified during the search strategy process (as shown in Appendix A). A senior librarian was consulted to assist with designing and refining search strings based on inclusion criteria. An initial search was conducted in March 2023 and a second search in March 2024 to identify any new articles that may have been published since the initial search. No authors were contacted for additional data.

### Study/ Source of evidence selection

Following the search, all identified citations were collated and uploaded into the *Mendeley Reference Management System* [40] and duplicates removed. From here, a.bibtex file was uploaded onto Rayyan [41], a web application for systematic reviews, scoping reviews, and literature, where a second detection of duplicates was undertaken.

Titles and abstracts were screened independently in triplicate by BT, EI and a research assistant for eligibility according to the inclusion criteria. The full texts of potentially relevant sources were further reviewed in duplicate (BT and EI) against inclusion criteria. Any disagreements between reviewers were resolved through discussion including an additional reviewer (PC). Reasons for exclusion at full text review were recorded.

### Data extraction and quality assessment

Data was extracted from included papers by two independent reviewers (BT and EI) according to specified table headings. The data extracted included specific details about the participants, concept, context, study methods and key findings relevant to the review questions. Any disagreements between the reviewers through discussion, or through an additional third reviewer (PC).

Quality assessment was conducted using the Newcastle-Ottawa Scale (NOS) [42]. This quality assessment scale was designed for non-randomised studies, specifically cohort- and case control studies. It has also been adapted for use with cross-sectional studies [43]. The relevant version of the NOS for each study type was used (appendix C). Two independent raters (LT and PC) assessed each article and a third (BT) moderated a consensus process.

## Results

**Fig. 1:**
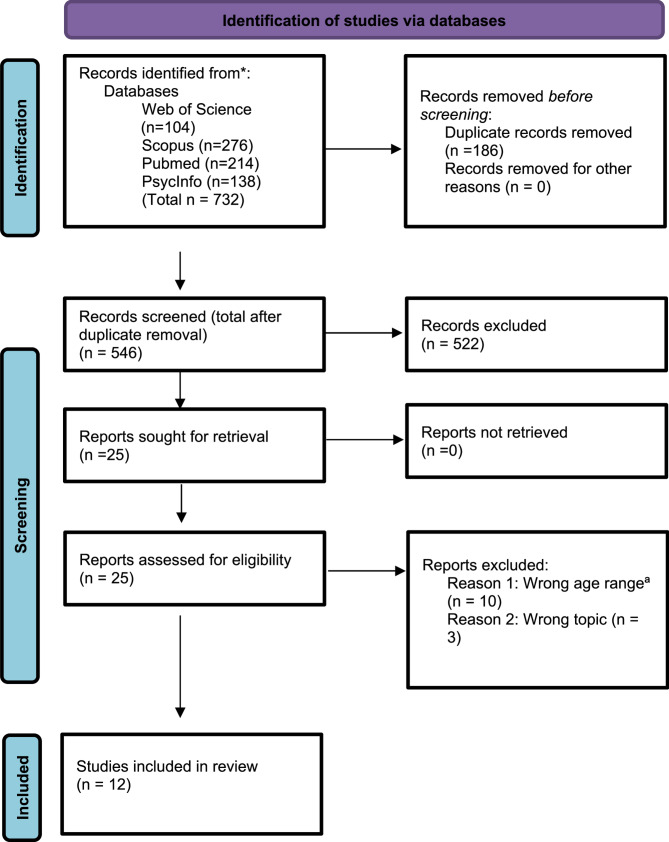
PRISMA-ScR flow diagram of search process. ª These articles overlapped with the age range 2- to 8 years but also included children between 0 and 2 years. Since reported findings were not stratified according to age, data for those 2 years and over could not be extracted. Therefore, these articles were excluded from this scoping review as the focus was not on infants

A total of 732 studies were found: 214 on PubMed; 104 on Web of Science; 276 on SCOPUS and 138 on PsycInfo. After duplicated articles were deleted, a total of 546 abstracts of studies were retained for further review. The results of the search and the study inclusion process are reported in a PRISMA-ScR flow diagram (Fig. 1) [38].

Twelve articles representing ten unique studies were included; six from South Africa, three from Kenya representing one large study, two from Uganda and one from Malawi. Of these, six articles included children age 9 years (range: 2–9 years or 6–9 years). It was decided to retain these articles as this age difference was not considered to meaningfully alter the extent of the group’s consistency with the research question. The focus remains on young children, as all children in these groups still fall below the period defined as adolescence by the WHO, which is 10 to 19 years [44].

Nine further studies were found that met all inclusion criteria except that children under the age of 2 years were also included, along with older children. The results of these studies were not age-disaggregated hence it was not possible to extract only the data pertaining to children aged 2–8 years. As infant neurodevelopment is more rapidly evolving and not the focus of our scoping review, these 9 studies were excluded. Some of these studies will nevertheless be referred to in the Discussion.

Two of the 12 included articles described screening explicitly for several types of NDDs [45, 46]. One formed part of a larger study that screened for various NDDs, but focused primarily on epilepsy [47]. Four articles focused on one specific NDD only. The remaining five screened for developmental delay only but considered several domains of functioning within this concept (e.g. gross motor, fine motor, communication, cognition, social-personal). The 12 articles were of a heterogeneous nature and are therefore individually summarised below (Table 1). 

Kakooza-Mwesige et al. [46] created the 23-Question Screener (23Q), using the existing WHO Ten Questions (TQ) screening questionnaire [48] with an additional 10 questions pertaining to ASD and 3 pertaining to hearing, vision and seizures. The 23Q was used with a sample of 1169 children between the ages of 2–9 years from a balanced urban-rural population in Uganda. Of these, 27% were screen-positive and received subsequent clinical and specialised assessments. Prevalence of 10–13/100 children for seven combined NDDs, including autism, was estimated. The 23Q demonstrated feasibility in a low-resource setting, with variable sensitivity (0.55–0.80) and specificity (0.77) depending on different assumptions. Despite challenges such as attrition and cultural factors impacting participation, the authors reported the screener as having potential for identifying children with NDDs in resource-limited settings.

Bitta et al. [45] sought to validate the use of the Neurodevelopmental Screening Tool (NDST) in Africa whilst establishing population prevalence within a large, two-stage epidemiological study in the Kilifi district in Kenya involving 11 223 children. The TQ was additionally employed. Those who screened positive and a portion of those who screened negative, were invited for comprehensive clinical- and neuropsychological assessments in the second stage of the study. The authors found high sensitivity (87.8%) and specificity (83.3%) on the NDST as a whole, concluding that it may be reliably used to screen for NDDs in a rural part of Africa. The NDST was considered preferable to the TQ because of its psychometric properties and inclusion of questions about ADHD and autism, thus screening for a broader array of NDDs. Trained interviewers, fluent in the local dialect, were used to administer the screens.

As part of the same large epidemiological study in Kilifi, Kenya, Kariuki et al. [49] conducted a comprehensive assessment of the efficacy of the ADHD module of the ‘Kiddie Schedule for Affective Disorders and Schizophrenia - Present and Lifetime Version’ (K-SADS-PL) in screening and diagnosing ADHD among children in Kenya. 2,074 children aged 6 to 9 years were screened using the ADHD module of the K-SADS-P, translated into Kiswahili. This was administered by assessors trained by a Child & Adolescent Psychiatrist, who also provided weekly supervision to assessors. 18.5% (385) reached the threshold and proceeded to further assessment using the diagnostic supplement of the ADHD K-SADS-PL module, and 77.4% of these received a diagnosis of ADHD. Minimal overlap with generalised anxiety disorder and substantial overlap with ODDs were found. The study reported excellent item reliability coefficients for both the screening and supplemental stages, and strong standardised coefficients. High sensitivities (~ 97%) and specificities (~ 95%) in the screening component supported the effectiveness of the ADHD K-SADS-PL module in identifying children in need of clinical assessment in resource-limited settings.

Kind et al. [47] focused on the prevalence of epilepsy as part of the same large Kenyan epidemiological study. Of the 11,223 children screened using the NDST, 21% screened positive for neurological disorders. Of these, 69.5% were seen for clinical assessments. Prevalence rates of 20.9 per 1,000 for lifetime epilepsy and 68.8 per 1,000 for acute symptomatic seizures, were established. Significantly, 70.4% of those eventually diagnosed with epilepsy screened positive for epilepsy; the remainder screened positive for other neurodevelopmental conditions such as autism and ADHD, on the NDST. Kariuki et al. [50] screened for acute seizures as part of this same large Kenyan epidemiological study, but this study was excluded from our results for the sake of consistency, due to the age range of included children having been 1 to 6 years.

In Uganda, Arinda et al. [51] used the Social Communication Questionnaire (SCQ) to screen for the possible presence of autism symptoms in children attending a paediatric neurology clinic. The principal investigator (a psychiatrist) and two research assistants (mental health nurses) administered the screens. Their findings showed that 45% of participants exhibited notable autism symptoms, with correlations found between delayed developmental milestones, speech difficulties, and the presence of autism symptoms.

In the domain of language development, Mazibuko and Chimbari [52] identified the need for home-language screening tools in the multilingual context of South Africa. They developed the Ingwavuma Receptive Vocabulary Test (IRVT) to screen for and assess receptive language in isiZulu. The IRVT was tested on children with a mean age of 5.7 years. It was identified that certain adjustments were required to better accommodate contextual and biopsychosocial factors.

Venter et al. [53] evaluated the psychometric properties of the Little Developmental Coordination Disorder Questionnaire (Little DCDQ) by screening, and then conducting gold standard assessments with, 53 children aged 3–5 years. They found good internal consistency (Chronbach’s Alpha, r = > 0.8), poor sensitivity (57.1%) and good specificity (81.3%).

Also in South Africa, Du Toit et al. [54] conducted a cross-sectional within-subject study where 276 children aged 36–83 months were screened for developmental delays using the Parental Evaluation of Developmental Status (PEDS) tools [55] in a smartphone-based mHealth application. The study took place in low-income communities affected by unemployment, poverty and undernutrition around the city of Tshwane. The aim was to validate the use of the mHealth-based PEDS by evaluating outcomes against a standard reference assessment tool, the Vineland-3. Eight domains of development were considered for possible delays. A sensitivity rate of 92.6% was found but specificity was only 22.5%. Considerations for specificity improvements were provided.

Two of the articles found screened for neurodevelopmental challenges in children with HIV. Devendra et al. [56] conducted a case-control study involving 296 children with HIV and their siblings without HIV. Disabilities were screened for using the TQ screener. The odds of children with HIV having a disability were found to be 8 times higher than those of their siblings without HIV. The study reports significant under-recognition and under-reporting of developmental disabilities prior to screening. Approximately half of all cases had two or more co-existing disabilities, highlighting the need for holistic, multi-disciplinary support services. Knox et al. [57] also used the TQ screener, translated into *isi*Zulu, to detect developmental disability in children with and without HIV in South Africa. 1787 children were screened at their homes. Of these, 1581 reported for a follow-up disability assessment by a medical doctor. The TQ was found to have high sensitivity for detecting serious developmental disabilities in children with and without HIV (100% and 90.2%), but low specificity (51.2%) and low positive predictive value (12.5%) among children with HIV. The study observed that HIV in children was associated with disability indicators such as delayed motor skills.

The impact of maternal wellbeing on early childhood development was the focus of a study conducted in South Africa by Shuffrey et al. [58]. Children born to mothers with increased exposure to ACEs and with depression and anxiety, were screened for cognitive-, language- and motor delays using the Bayley Scales of Infant Development III - Screening Test. Lower cognitive scores were found among children born to mothers with concurrent prenatal depression and trait anxiety. Additionally, increased socioemotional problems were found in the same group. No further assessment to confirm screening outcomes were reported.

Brittain et al. [59] also focused on maternal psychosocial challenges. Conducted within a larger study longitudinally following pregnant women with HIV, 266 children were assessed at 36–60 months follow-up using the Ages & Stages Questionnaire: Third Edition (ASQ-3). Here, maternal ACEs were associated with poorer socioemotional development in children, but not with impaired functioning on 5 neurodevelopmental domains. No further assessment to confirm screening outcomes were reported.

Quality assessment using the NOS yielded ratings ranging from “Very good” to “Unsatisfactory” (cross-sectional studies) and “Good” to “Fair” (cohort- and case-control studies) (Appendix D).

**Table 1:** Data Extraction Summary of 12 Articles Utilising Neurodevelopmental Screening Tools in sub-Saharan Africa.

Author, year of publication, country	Study design and setting		NDDs being screened for	Screening tool used	Who administered screening?	Sample size and participants		Method of outcome measurement
Kakooza-Mwesige et al., 2014, Uganda	Cross-sectional. Assessed the clinical validity of an adapted screening tool. Population-wide sampling through household screening in rural and urban parishes in Uganda selected through cluster sampling.		Cognitive and motor impairment, seizure disorders, autism, serious speech-, vision- and hearing impairments	23Q consisting of WHO TQ, 10 additional ASD questions and 3 questions about vision, hearing, and seizures. *Translated into Luganda.*	Research assistants, initially accompanied by ‘parish mobilisers’ (individuals known in community who helped facilitate participation)	1169 children aged 2–9 years		Clinical assessment by a medical officer blinded to screening outcomes. Children with suspected disabilities were referred to experienced specialists for assessment and diagnosis.
Bitta et al., 2021, Kenya	Population-wide, cross-sectional, two-stage epidemiological study. Screening of randomly selected children followed by detailed assessments. Conducted in Kilifi County, Kenya.		Presence of NDDs including ADHD, autism, epilepsy and cognitive impairment.	NDST TQ *Translated into Kiswahili.*	Trained interviewers fluent in the local dialects conducted home visits to explain the study to parents, obtain informed consent and administer screening questionnaires	11,223 children aged 6–9 years living in the Kilifi area		Clinical history and neuro-psychological assessments – several specialist assessments (e.g. ADOS, Ravens Matrix) according to relevant problem areas.
Kariuki et al., 2020, Kenya	Cross-sectional study nested within a large population-wide epidemiological study (using NDST) in Kilifi, Kenya. Assessed reliability of ADHD-module of K-SADS-PL to screen for ADHD.		ADHD	ADHD module of the K-SADS-PL – used in interview with parents	Neuropsychological assessors and primary health care workers trained and supervised by a Child & Adolescent Psychiatrist	2,074 children aged 6–9 in the community		Supplement ADHD module of K-SADS-PLK. Clinical assessment by blinded Child & Adolescent Psychiatrist for a sample of 20 children with and without ADHD.
Kind et al., 2017, Kenya	2-stage cross-sectional population-wide study nested within large two-stage epidemiological study in Kilifi.		Epilepsy	NDST *Translated into Kiswahili*		11,223 randomly selected children aged 6–9		Clinical assessment EEG Risk factor questionnaire.
Arinda et al., 2020, Uganda	Cross-sectional study assessing the prevalence of autism symptoms among children attending the paediatric neurology outpatient clinic at Mulago Hospital, Uganda.		Autism spectrum disorder	SCQ	Administered by the researcher (a psychiatrist) and two mental health nurses.	318 children aged 2–9 years attending the paediatric neurology clinic of Mulago Hospital.		No further assessment (screen positives were directed to the child and adolescent mental health clinic for further assistance).
Mazibuko & Chimbari, 2020, South Africa	Cross-sectional study using children from general population; descriptive in nature. Developing and evaluating a new screening tool for use in rural isiZulu-speaking part of South Africa.		Receptive language skills	Ingwavuma Receptive Vocabulary Test *(newly developed tool in isiZulu)*	Clinicians	51 children aged 4 - <7 monolingual isiZulu speakers attending isiZulu medium pre-schools around a rural village		None
Venter et al., 2015, South Africa	Cross-sectional validation study, evaluating reliability and validity of screening tool		Develop-mental coordination disorder	Little DCDQ	Questionnaires completed by parents at home; illiterate parents were requested to visit school where translators helped them complete the questionnaires	53 children age 3–5 years attending 5 pre-schools in the area		Movement Assessment Battery for Children − 2 as gold standard assessment
Du Toit et al., 2021, South Africa	Cross-sectional study screening pre-school children from low-income communities around the City of Tshwane, selected through stratified random sampling.		Develop-mental delay (included 8 domains of functioning)	mHealth PEDS developmental screening	Smartphone application completed by caregivers.	276 children aged 36–83 months attending ECDs in identified area.		Vineland-3 (Comprehensive caregiver form completed by carers at home; collected ± 1 week later from ECD centres)
Devendra et al., 2013, Malawi	Case-controlled study with children selected from a paediatric anti-retroviral centre in Lilongwe, Malawi.		Presence and types of disability	WHO TQ	Trained study assistant	296 children with HIV aged 2–9 attending HIV treatment services and their siblings without HIV as paired controls.		No clinical assessment to confirm disability Clinical data extracted from electronic medical records if available.
Knox et al., 2018, South Africa		Cross-sectional observation study nested in the larger, longitudinal Asenze study in the Kwa-Zulu Natal province, characterised by peri-urban dwellings, high HIV rates, food insecurity and unemployment	Develop-mental disability	TQ *Translated into isiZulu*	Not specified	1, 787 children aged 4–6 years identified through household visits.		Disability assessment conducted by a medical doctor Reynell assessment for language delay and Grover Counter test administered by independent psychological assessors
Shuffrey et al., 2021, South Africa		Subset of data from the multi-centre ‘Safe Passage’ prospective cohort study, examining the association between prenatal maternal mental health and child neuro-development at age 3 years.	Child social-­emotional and cognitive develop-ment	BSID-­III ST Brief Infant­ Toddler Social Emotional Assessment	Not specified	600 maternal-infant pairs	Outcome on screening instrument was considered indicative of outcome	
Brittain et al., 2022, South Africa		Cohort study at a large primary care clinic in a peri-urban township near Cape Town. Part of larger ‘MCH-ART’ study on supporting women living with HIV through pregnancy and post-partum.	Five domains of child deve-lopment and socio-emotional develop-ment	ASQ-3 ASQ-SE *Translated into isiXhosa*	Not specified	353 women living with HIV attended the study follow-up visit after child was born; 266 attended with their child.	Outcome on screening instrument was considered indicative of outcome	

*Abbreviations*: *23Q*, 23 Item Questionnaire, *ADOS *Autism Diagnostic Observation Schedule, *ASQ-3* Ages & Stages Questionnaire – Third Edition, *BSID-III ST *Bayley Scales of Infant Development Third Edition Screening Test, *DCDQ * Developmental Coordination Disorder Questionnaire, *ECD *Early Childhood Development, *K-SADS-PL * Kiddie Schedule for Affective Disorders and Schizophrenia - Present and Lifetime Version, *NDD * Neurodevelopmental Disorders, *NDST* Neurodevelopmental Screening Tool,* PEDS* Parent’s Evaluation of Developmental Status, *SCQ* Social Communication Questionnaire, *TQ* Ten Questions Questionnaire, *WHO* World Health Organisation.

## Discussion

This scoping review examined existing research on the use of neurodevelopmental screeners amongst children aged 2- to 8-years in sSA. Twelve articles representing 10 distinct studies were found. A variety of screening tools were used, screening for either one specific type of NDD, general developmental delay across different developmental domains, or for a variety of NDDs. Many made use of trained assistants for screening, while researchers themselves conducted screening in others, or parents completed questionnaires at home. Population-wide screening has been undertaken in some population groups in certain countries (notably, 45, 46).

That only 12 articles were found published since 2010 that met our search criteria, supports the findings of a systematic review and meta-analysis conducted by Rah et al. [60] who comprehensively clarified the need for more screening studies in LMIC as well as in marginalised groups within high-income countries. The published articles found in the present scoping review were from only 4 of the 46 countries in sSA - South Africa, Kenya, Uganda, and Malawi. While this will have been influenced by language of publication, evidence related to screening for NDDs in early childhood appear to occur in ‘pockets’ across various populations in sSA. We found great variability regarding the NDDs screened for. Most studies screened for specific NDDs or general delays, whereas three used instruments that screen for a range of NDDs.

The variation in quality assessment ratings obtained using the NOS, should be considered against the fact that most of the assessed studies focused on the creation of new knowledge in relatively under-researched fields, or on the validation of a screener in particular contexts. There may therefore be limitations to the comprehensive adequacy of the NOS outcomes in reflecting the value and relevance of these studies. Several of the studies did not explicitly indicate the comparability of respondents and non-respondents in the event of unsatisfactory response rate, or explicitly establish comparability of outcome groups (through study design or analysis that control for confounding factors). However, most of the reviewed studies are aligned with recommendations that NDD screening processes in sSA should be contextually appropriate and linguistically accessible. As such, these studies collectively fulfil a crucial need.

Validation of screening instruments for use in the local context, as well as contextually specific adaptations of the screening process, emerged as important themes in these articles. Some aimed to validate specific or adapted screeners; some created new screeners; and several translated existing screeners that are already used in other countries, into local languages. Kakooza-Mwesige et al. [46] describe processes of extensive community consultation and involvement engaged in prior to rolling out a screening project. They described context-specific challenges including challenges for parents to attend follow-up assessments as travel distances required time to be taken off from work. This highlights the barriers, in many parts of sSA, to accessing service-delivery settings where NDD screening and sustained follow-up services are undertaken.

In several of the articles in our sample, screening did not form part of a greater programme of accessible, multi-disciplinary diagnostic and intervention services. Resource constraints played a role in this regard in some studies and is mostly a factor requiring considerable ongoing development across the region. However, in some cases screening did lead to further clinical assessment and possible diagnoses of an identifiable NDD. Kakooza-Mwesige et al. [46] followed screening for seven NDDs with detailed assessment. Bitta et al. [45] and Kind et al. [45] employed a similar two-stage model, reflecting a move towards broader neurodevelopmental screening across a range of NDDs.

Depending on the focus of the investigation, some studies evaluated screens against a standard reference assessment, for example the Vineland-3 in Du Toit et al. [54]. Others referred those that screened positive onwards to local clinics for further assessment, but such further support trajectories and their outcomes were not described further (e.g. 51).

Some of the studies provided specific information regarding who administered the screening and what support they were given [49, 51]. Information about the precise extent of initial training provided to screening personnel was generally limited. These are important considerations, due to the relative shortage of trained medical professionals and the many cultural, linguistic and socio-economic variables across sSA. Training community care workers who are not health professionals to conduct screening, can increase the capacity for larger scale screening projects in the region.

Using of existing screening instruments on a smartphone-based platform - such as the mHealth PEDS [54] - offers promising avenues for improving accessibility in underdeveloped areas. mHealth has been widely used and researched in South Africa [61] for a variety of purposes. In neurodevelopmental screening, increased accessibility remains a potential advantage of this medium.

Screening tools are valuable resources for healthcare professionals, educators, and caregivers. They aid in the identification of potential concerns during critical growth periods and enable prompt intervention and support [10, 54]. Importantly, screening for NDDs in young children aligns with the growing consensus favouring holistic responses to childhood development needs [13, 21, 23]. Effective and appropriate neurodevelopmental screening requires a clear concept of who should be screened, for what they should be screened, when, and by whom. Clarification of these elements of screening for NDDs in sSA requires a significant amount of further study.

There has been a notable lack of emphasis on recognising and strategizing for developmental concerns in children in Africa [54, 62–64]. A critical challenge lies in the limited research validation of practical toolsets in this context - particularly in regions where cultural and linguistic factors impact on the applicability of existing tools [52]. Some screening for NDDs has started to take place in certain parts of sSA, and awareness of NDDs is growing in some specific clinical settings in the region [49]. Important themes emerging from existing research include the importance of community involvement and -consultation in preparation for screening; sensitivity to linguistic and cultural factors; finding creative ways to improve accessibility of services; and optimising professional resources by training assessors who may be supervised by senior clinicians.

Such factors received considerable attention, too, in some of the articles we excluded due to the age range of participating children, which overlapped with ours but included infants. Botes et al. [65] describe robust community involvement in considering the cultural and linguistic appropriateness of the PEDS screening instrument for children aged 0 to 8 years in South Africa. Chambers et al. [66] describe extensive preparation and foundational work to ensure community support for, as well as participation in, assessing the cultural appropriateness of early autism detection tools amongst children aged 12 to 48 months in an *isi*Zulu community in South Africa. Translation of an Autism screener into another South African language, Northern Sotho, was the focus ofVorster et al. [67, 68], who used the Modified Checklist for Autism in Toddlers (M-CHAT) with children aged 18 to 48 months.

This scoping review highlights pragmatic challenges to overcome in undertaking screening of NDDs amongst young children in under-resourced sSA communities, including ensuring the contextual and linguistic appropriateness of screening tools used and ensuring community involvement.

Limitations of this study include the fact that our search parameters – including age limits, search terms used, and searching four large English-language databases only - may have led to some appropriate studies being missed. It is recognised that a search of studies screening for NDDs across wider age ranges, to potentially identify studies with data aggregated by age, may have added additional information to this scoping review.

### Implications for further research

Further research to validate rapid, reliable, contextually appropriate NDD screening instruments in sSA is needed. Screening practice which considers all the signs and symptoms of neurodevelopmental challenges associated with NDDs, would support the early detection of a wider range of NDDs. Screening for only one area of neurodevelopment risks missing potential other co-occurring neurodevelopmental conditions. Appropriate, effective screening is the first step to provide population-based prevalence data which may in turn inform public health policy, including resource allocation at government level, for the development of comprehensive diagnostic and support services.

## Supplementary Information

Supplementary Material 1.

## Data Availability

No datasets were generated or analysed during the current study.

## Abbreviations

23Q: 23 Item Questionnaire
ACE: Adverse Childhood Events
ADOS: Autism Diagnostic Observation Schedule
ADHD: Attention-deficit/hyperactivity disorder
ASQ-3: Ages & Stages Questionnaire – Third Edition
BSID-III ST: Bayley Scales of Infant Development Third Edition Screening Test
CD: Conduct Disorder
DCD: Developmental Coordination Disorder
DCDQ: Developmental Coordination Disorder Questionnaire
ECD: Early Childhood Development
ESSENCE: Early Symptomatic Syndromes Eliciting Neurodevelopmental Clinical Examinations
ID: Intellectual Developmental Disorder
IRVT: Ingwavuma Receptive Vocabulary Test
K-SADS-PL: Kiddie Schedule for Affective Disorders and Schizophrenia - Present and Lifetime Version
LIMC: Low- and middle-income countries
NDDs: Neurodevelopmental disorders
NDST: Neurodevelopmental Screening Tool
NOS: Newcastle-Ottawa Scale
ODD: Oppositional Defiant Disorder
PANDAS: Pediatric autoimmune neuropsychiatric disorders associated with Streptococcus infections
PANS: Pediatric Acute-onset Neuropsychiatric Syndrome
PEDS: Parent’s Evaluation of Developmental Status
PRISMA-ScR: Preferred Reporting Items for Systematic Reviews and Meta-Analyses - Extension for Scoping Reviews
SCQ: Social Communication Questionnaire
sSA: sub-Saharan Africa
TQ: Ten Questions Questionnaire
WHO: World Health Organisation
