# Supplementary material for: Screening young children for neurodevelopmental differences in sub-Saharan Africa: a scoping review
Source: BMC Psychiatry. 2025 Sep 15;25:857. doi: 10.1186/s12888-025-07279-0 (PMC12439364; doi:10.1186/s12888-025-07279-0)
Supplement: Supplementary file 1 — Supplementary Material 1. [file 12888_2025_7279_MOESM1_ESM.pdf]

Appendix A

Table. PRISMA-ScR Checklist

Section	Item	PRISMA-ScR Checklist Item
**Title**	1	Identify the report as a scoping review.
**Abstract**		
Structured summary	2	Provide a structured summary that includes (as applicable) background, objectives, eligibility criteria, sources of evidence, charting methods, results, and conclusions that relate to the review questions and objectives.
**Introduction**		
Rationale	3	Describe the rationale for the review in the context of what is already known. Explain why the review questions/objectives lend themselves to a scoping review approach.
Objectives	4	Provide an explicit statement of the questions and objectives being addressed with reference to their key elements (e.g., population or participants, concepts, and context) or other relevant key elements used to conceptualize the review questions and/or objectives.
**Methods**		
Protocol and registration	5	Indicate whether a review protocol exists; state if and where it can be accessed (e.g., a Web address); and if available, provide registration information, including the registration number.
Eligibility criteria	6	Specify characteristics of the sources of evidence used as eligibility criteria (e.g., years considered, language, and publication status), and provide a rationale.
Information sources*	7	Describe all information sources in the search (e.g., databases with dates of coverage and contact with authors to identify additional sources), as well as the date the most recent search was executed.
Search	8	Present the full electronic search strategy for at least 1 database, including any limits used, such that it could be repeated.
Selection of sources of evidence†	9	State the process for selecting sources of evidence (i.e., screening and eligibility) included in the scoping review.
Data charting process‡	10	Describe the methods of charting data from the included sources of evidence (e.g., calibrated forms or forms that have been tested by the team before their use, and whether data charting was done independently or in duplicate) and any processes for obtaining and confirming data from investigators.
Data items	11	List and define all variables for which data were sought and any assumptions and simplifications made.
Critical appraisal of individual sources of evidence§	12	If done, provide a rationale for conducting a critical appraisal of included sources of evidence; describe the methods used and how this information was used in any data synthesis (if appropriate).
Summary measures	13	Not applicable for scoping reviews.
Synthesis of results	14	Describe the methods of handling and summarizing the data that were charted.
Risk of bias across studies	15	Not applicable for scoping reviews.
Additional analyses	16	Not applicable for scoping reviews.
**Results**		
Selection of sources of evidence	17	Give numbers of sources of evidence screened, assessed for eligibility, and included in the review, with reasons for exclusions at each stage, ideally using a flow diagram.
Characteristics of sources of evidence	18	For each source of evidence, present characteristics for which data were charted and provide the citations.
Critical appraisal within sources of evidence	19	If done, present data on critical appraisal of included sources of evidence (see item 12).
Results of individual sources of evidence	20	For each included source of evidence, present the relevant data that were charted that relate to the review questions and objectives.
Synthesis of results	21	Summarize and/or present the charting results as they relate to the review questions and objectives.
Risk of bias across studies	22	Not applicable for scoping reviews.
Additional analyses	23	Not applicable for scoping reviews.
**Discussion**		
Summary of evidence	24	Summarize the main results (including an overview of concepts, themes, and types of evidence available), link to the review questions and objectives, and consider the relevance to key groups.
Limitations	25	Discuss the limitations of the scoping review process.
Conclusions	26	Provide a general interpretation of the results with respect to the review questions and objectives, as well as potential implications and/or next steps.
**Funding**	27	Describe sources of funding for the included sources of evidence, as well as sources of funding for the scoping review. Describe the role of the funders of the scoping review.

JBI = Joanna Briggs Institute; PRISMA-ScR = Preferred Reporting Items for Systematic reviews and Meta-Analyses extension for Scoping Reviews.
* Where *sources of evidence* (see second footnote) are compiled from, such as bibliographic databases, social media platforms, and Web sites.
† A more inclusive/heterogeneous term used to account for the different types of evidence or data sources (e.g., quantitative and/or qualitative research, expert opinion, and policy documents) that may be eligible in a scoping review as opposed to only studies. This is not to be confused with *information sources* (see first footnote).
‡ The frameworks by Arksey and O'Malley (6) and Levac and colleagues (7) and the JBI guidance (4, 5) refer to the process of data extraction in a scoping review as data charting.
§ The process of systematically examining research evidence to assess its validity, results, and relevance before using it to inform a decision. This term is used for items 12 and 19 instead of "risk of bias" (which is more applicable to systematic reviews of interventions) to include and acknowledge the various sources of evidence that may be used in a scoping review (e.g., quantitative and/or qualitative research, expert opinion, and policy documents).

From: Tricco AC, Lillie E, Zarin W, O'Brien KK, Colquhoun H, Levac D, et al. PRISMA Extension for Scoping Reviews (PRISMAScR): Checklist and Explanation. Ann Intern Med. 2018;169:467–473. doi: 10.7326/M18-0850.

Appendix B

PubMed Search Strategy 2024/03

#	Search	Results
#1	"Neurologic Manifestations "[Mesh] OR "Neurodevelopmental Disorders"[Mesh] OR "Fetal Alcohol Spectrum Disorders"[Mesh]	1,442,485
#2	"Pediatric Autoimmune Neuropsychiatric Disorders Associated with Streptococcal infections"[Text Word] OR "Pediatric acute-onset neuropsychiatric syndrome"[Text Word] OR "PANS"[Text Word] OR neuropsychiatr*[Text Word] OR "sensory dysregulation"[Text Word] OR "developmental delay*"[Text Word] OR "developmental disability*"[Text Word] OR "developmental concern*"[Text Word] OR "brain development"[Text Word] OR "cognitive development*"[Text Word] OR "neural development"[Text Word] OR neurodevelopment*[Text Word] NOT hearing[Text Word] NOT ocular[Text Word] NOT vision[Text Word]	148,199
#3	#1 OR #2	1,552,849
#4	"Africa, Southern"[Mesh] OR "Africa South of the Sahara"[Mesh]	266,319
#5	Angola[Text Word] OR Benin[Text Word] OR Botswana[Text Word] OR Bobo-Dioulasso[Text Word] OR "Burkina Faso"[Text Word] OR Burundi[Text Word] OR Cameroon[Text Word] OR "Cape Verde"[Text Word] OR "Central African Republic"[Text Word] OR Chad[Text Word] OR Comoros[Text Word] OR Congo[Text Word] OR Brazzaville[Text Word] OR "Cote d'Ivoire"[Text Word] OR "Ivory Coast"[Text Word] OR Djibouti[Text Word] OR "Equatorial Guinea"[Text Word] OR Eritrea[Text Word] OR Ethiopia[Text Word] OR Gabon[Text Word] OR Gambia[Text Word] OR Ghana[Text Word] OR Guinea[Text Word] OR Bissau[Text Word] OR Kenya[Text Word] OR Lesotho[Text Word] OR Liberia[Text Word] OR Madagascar[Text Word] OR Malawi[Text Word] OR Mali[Text Word] OR Mauritania[Text Word] OR Mauritius[Text Word] OR Mozambique[Text Word] OR Namibia[Text Word] OR Niger[Text Word] OR Nigeria[Text Word] OR Rwanda[Text Word] OR "Sao Tome e Principe"[Text Word] OR Senegal[Text Word] OR Seychelles[Text Word] OR "Sierra Leone"[Text Word] OR Somalia[Text Word] OR "South Africa"[Text Word] OR South Sudan[Text Word] OR Sudan[Text Word] OR Swaziland[Text Word] OR Tanzania[Text Word] OR Togo[Text Word] OR Uganda[Text Word] OR Western Sahara[Text Word] OR Zaire[Text Word] OR Zambia[Text Word] OR Zimbabwe[Text Word] OR "Africa South of the Sahara"[Text Word] OR "Sub-Saharan Africa"[Text Word] OR "Southern Africa"[Text Word] OR "Subsaharan Africa*"[Text Word] OR "sub-sahara*"[Text Word] OR "subsahara*"[Text Word]	541,896
#6	#4 OR #5	548,567

#7	"mass screening"[Mesh]	145,347
#8	screen*[Text Word] OR "screening tool*"[Text Word]	1,101,000
#9	#7 OR #8	1,110,616
#10	"child"[MeSH Terms]	2,191,215
#11	"child*", "young children", infant, toddler, "Grade R"[Text Word] OR "Grade 1"[Text Word] OR "First Grade"[Text Word] OR "elementary school*"[Text Word] OR kindergarten*[Text Word] OR preschool[Text Word] OR pre-school[Text Word] OR "pre-primary school"[Text Word] OR "Gr 2"[Text Word] OR "Grade 2"[Text Word]	1,066,042
#12	#10 OR #11	2,247,688
#13	("2012/01/01"[Date - Publication] : "2023/03/01"[Date - Publication])	13,889,982
#14	#3 AND #6 AND #9 AND #12 AND #13	214

Web of Science Searching Strategy 2024/03

#	Search	Results
#1	"Pediatric Autoimmune Neuropsychiatric Disorders Associated with Streptococcal infections" OR PANDAS OR "Pediatric acute-onset neuropsychiatric syndrome" OR "PANS" OR neuropsychiatr* OR "sensory dysregulation" OR "developmental delay*" OR "developmental disability*" OR "developmental concern*" OR "brain development" OR "cognitive development*" OR "neural development" OR neurodevelopment* NOT hearing NOT ocular NOT vision (Topic)	181,394

#2	Angola OR Benin or Botswana OR Bobo-Dioulasso OR “Burkina Faso” OR Burundi OR Cameroon OR “Cape Verde” OR “Central African Republic” OR Chad OR Comoros OR Congo OR Brazzaville OR “Cote d'Ivoire” OR “Ivory Coast” OR Djibouti OR “Equatorial Guinea” OR Eritrea OR Ethiopia OR Gabon OR Gambia OR Ghana OR Guinea OR Bissau OR Kenya OR Lesotho OR Liberia OR Madagascar OR Malawi OR Mali OR Mauritania OR Mauritius OR Mozambique OR Namibia OR Niger OR Nigeria OR Rwanda OR “Sao Tome e Principe” OR Senegal OR Seychelles OR “Sierra Leone” OR Somalia OR “South Africa” OR South Sudan OR Sudan OR Swaziland OR Tanzania OR Togo OR Uganda OR Western Sahara OR Zaire OR Zambia OR Zimbabwe OR "Africa South of the Sahara" or "Sub-Saharan Africa" OR “Southern Africa” OR "Subsaharan Africa*" OR "sub-sahara*" OR "subsahara*" (Topic)	795,082
#3	"mass screening" OR screen* OR "screening tool*" (Topic)	1,216,285
#4	child* OR “young children” OR infant OR toddler OR “Grade R" OR "Grade 1" OR "First Grade" OR "elementary school*" OR kindergarten* OR preschool OR pre-school OR "pre-primary school" OR "Gr 2" OR "Grade 2" (Topic)	2,583,691
#5	#1 AND #2 AND #3 AND #4 AND 2012-01-01 to 2023-03-01 (Publication Date)	104

PsycInfo Scoping Searching Strategy 2024/03

#	Search	Results
#1	"Pediatric Autoimmune Neuropsychiatric Disorders Associated with Streptococcal infections" OR PANDAS OR "Pediatric acute-onset neuropsychiatric syndrome" OR "PANS” OR neuropsychiatr* OR "sensory dysregulation" OR "developmental delay*" OR "developmental disability*" OR "developmental concern*" OR "brain development" OR "cognitive development*" OR "neural development" OR neurodevelopment* NOT hearing NOT ocular NOT vision (Topic)	220,788

#2	Angola OR Benin or Botswana OR Bobo-Dioulasso OR “Burkina Faso” OR Burundi OR Cameroon OR “Cape Verde” OR “Central African Republic” OR Chad OR Comoros OR Congo OR Brazzaville OR “Cote d'Ivoire” OR “Ivory Coast” OR Djibouti OR “Equatorial Guinea” OR Eritrea OR Ethiopia OR Gabon OR Gambia OR Ghana OR Guinea OR Bissau OR Kenya OR Lesotho OR Liberia OR Madagascar OR Malawi OR Mali OR Mauritania OR Mauritius OR Mozambique OR Namibia OR Niger OR Nigeria OR Rwanda OR “Sao Tome e Principe” OR Senegal OR Seychelles OR “Sierra Leone” OR Somalia OR “South Africa” OR South Sudan OR Sudan OR Swaziland OR Tanzania OR Togo OR Uganda OR Western Sahara OR Zaire OR Zambia OR Zimbabwe OR "Africa South of the Sahara" or "Sub-Saharan Africa" OR “Southern Africa” OR "Subsaharan Africa*" OR "sub-sahara*" OR "subsahara*" (Topic)	89,628
#3	"mass screening" OR screen* OR "screening tool*" (Topic)	155,014
#4	child* OR “young children” OR infant OR toddler OR “Grade R" OR "Grade 1" OR "First Grade" OR "elementary school*" OR kindergarten* OR preschool OR pre-school OR "pre-primary school" OR "Gr 2" OR "Grade 2" (Topic)	1,186,432
#5	#1 AND #2 AND #3 AND #4 AND 2012 to 2023 (Publication Date)	138

SCOPUS Searching Strategy 2024/03

#	Search	Results
#6	(TITLE-ABS-KEY ("Pediatric Autoimmune Neuropsychiatric Disorders Associated with Streptococcal infections" OR pandas OR "Pediatric acute-onset neuropsychiatric syndrome" OR "PANS" OR neuropsychiatr* OR "sensory dysregulation" OR "developmental delay*" OR "developmental disability*" OR "developmental concern*" OR "brain development" OR "cognitive development*" OR "neural development" OR neurodevelopment* AND NOT hearing AND NOT ocular AND NOT vision)) AND (TITLE-ABS-KEY ("mass screening" OR screen* OR "screening tool*")) AND (TITLE-ABS-KEY (child* OR "young children" OR infant OR toddler OR "Grade R" OR "Grade 1" OR "First Grade" OR "elementary school*" OR kindergarten* OR preschool OR pre-school OR "pre-primary school" OR "Gr 2" OR "Grade 2")) AND (LIMIT-TO (PUBYEAR , 2023) OR LIMIT-TO (PUBYEAR , 2022) OR LIMIT-TO (PUBYEAR , 2021) OR LIMIT-TO (PUBYEAR , 2020) OR LIMIT-TO (PUBYEAR , 2019) OR LIMIT-TO (PUBYEAR , 2018) OR LIMIT-TO (PUBYEAR , 2017) OR LIMIT-TO (PUBYEAR , 2016)	276

	OR LIMIT-TO (PUBYEAR , 2015) OR LIMIT-TO (PUBYEAR , 2014) OR LIMIT-TO (PUBYEAR , 2013) OR LIMIT-TO (PUBYEAR , 2012)) AND (LIMIT-TO (AFFILCOUNTRY , "South Africa") OR LIMIT-TO (AFFILCOUNTRY , "Nigeria") OR LIMIT-TO (AFFILCOUNTRY , "Kenya") OR LIMIT-TO (AFFILCOUNTRY , "Uganda") OR LIMIT-TO (AFFILCOUNTRY , "Ethiopia") OR LIMIT-TO (AFFILCOUNTRY , "Tanzania") OR LIMIT-TO (AFFILCOUNTRY , "Ghana") OR LIMIT-TO (AFFILCOUNTRY , "Botswana") OR LIMIT-TO (AFFILCOUNTRY , "Zambia") OR LIMIT-TO (AFFILCOUNTRY , "Congo") OR LIMIT-TO (AFFILCOUNTRY , "Democratic Republic Congo") OR LIMIT-TO (AFFILCOUNTRY , "Zimbabwe") OR LIMIT-TO (AFFILCOUNTRY , "Mozambique") OR LIMIT-TO (AFFILCOUNTRY , "Rwanda") OR LIMIT-TO (AFFILCOUNTRY , "Benin") OR LIMIT-TO (AFFILCOUNTRY , "Malawi") OR LIMIT-TO (AFFILCOUNTRY , "Sudan") OR LIMIT-TO (AFFILCOUNTRY , "Cameroon") OR LIMIT-TO (AFFILCOUNTRY , "Gabon") OR LIMIT-TO (AFFILCOUNTRY , "Angola") OR LIMIT-TO (AFFILCOUNTRY , "Gambia") OR LIMIT-TO (AFFILCOUNTRY , "Liberia") OR LIMIT-TO (AFFILCOUNTRY , "Burkina Faso") OR LIMIT-TO (AFFILCOUNTRY , "Central African Republic") OR LIMIT-TO (AFFILCOUNTRY , "Guinea") OR LIMIT-TO (AFFILCOUNTRY , "Madagascar") OR LIMIT-TO (AFFILCOUNTRY , "Mali") OR LIMIT-TO (AFFILCOUNTRY , "Mauritania") OR LIMIT-TO (AFFILCOUNTRY , "Senegal") OR LIMIT-TO (AFFILCOUNTRY , "Sierra Leone") OR LIMIT-TO (AFFILCOUNTRY , "Somalia") OR LIMIT-TO (AFFILCOUNTRY , "Togo"))	
#5	(TITLE-ABS-KEY ("Pediatric Autoimmune Neuropsychiatric Disorders Associated with Streptococcal infections" OR pandas OR "Pediatric acute-onset neuropsychiatric syndrome" OR "PANS" OR neuropsychiatr* OR "sensory dysregulation" OR "developmental delay*" OR "developmental disability*" OR "developmental concern*" OR "brain development" OR "cognitive development*" OR "neural development" OR neurodevelopment* AND NOT hearing AND NOT ocular AND NOT vision)) AND (TITLE-ABS-KEY ("mass screening" OR screen* OR "screening tool*")) AND (TITLE-ABS-KEY (child* OR "young children" OR infant OR toddler OR "Grade R" OR "Grade 1" OR "First Grade" OR "elementary school*" OR kindergarten* OR preschool OR pre-school OR "pre-primary school" OR "Gr 2" OR "Grade 2")) AND (LIMIT-TO (PUBYEAR , 2023) OR LIMIT-TO (PUBYEAR , 2022) OR LIMIT-TO (PUBYEAR , 2021) OR LIMIT-TO (PUBYEAR , 2020) OR LIMIT-TO (PUBYEAR , 2019) OR LIMIT-TO (PUBYEAR , 2018) OR LIMIT-TO (PUBYEAR , 2017) OR LIMIT-TO (PUBYEAR , 2016) OR LIMIT-TO (PUBYEAR , 2015) OR LIMIT-TO (PUBYEAR , 2014) OR LIMIT-TO (PUBYEAR , 2013) OR LIMIT-TO (PUBYEAR , 2012))	7, 380
#4	(TITLE-ABS-KEY ("Pediatric Autoimmune Neuropsychiatric Disorders Associated with Streptococcal infections" OR pandas OR "Pediatric acute-onset neuropsychiatric syndrome" OR "PANS" OR neuropsychiatry* OR "sensory dysregulation" OR "developmental delay*" OR "developmental disability*" OR "developmental concern*" OR "brain development" OR "cognitive development*" OR "neural development" OR neurodevelopment* AND NOT hearing AND NOT ocular AND NOT vision)) AND (TITLE-ABS-KEY ("mass screening" OR screen* OR "screening tool*")) AND (TITLE-ABS-KEY (child* OR "young children" OR infant OR toddler OR "Grade R" OR "Grade	9,606

	1" OR "First Grade" OR "elementary school*" OR kindergarten* OR preschool OR pre-school OR "pre-primary school" OR "Gr 2" OR "Grade 2"))	
#3	(child* OR "young children" OR infant OR toddler OR "Grade R" OR "Grade 1" OR "First Grade" OR "elementary school*" OR kindergarten* OR preschool OR pre-school OR "pre-primary school" OR "Gr 2" OR "Grade 2")	4,601,244
#2	TITLE-ABS-KEY ("mass screening" OR screen* OR "screening tool*")	1,973,169
#1	TITLE-ABS-KEY ("Pediatric Autoimmune Neuropsychiatric Disorders Associated with Streptococcal infections" OR pandas OR "Pediatric acute-onset neuropsychiatric syndrome" OR "PANS" OR neuropsychiatr* OR "sensory dysregulation" OR "developmental delay*" OR "developmental disability*" OR "developmental concern*" OR "brain development" OR "cognitive development*" OR "neural development" OR neurodevelopment* AND NOT hearing AND NOT ocular AND NOT vision)	373,058

APPENDIX C

i. NEWCASTLE-OTTAWA SCALE ADAPTED FOR CROSS-SECTIONAL STUDIES

Note*: A study can be awarded a maximum of one star for each numbered item within the Selection and Outcome categories. A maximum of two stars can be given for Comparability.*

Selection

1. Representativeness of the sample
   a. Truly representative of the average in the target population ★ (random sampling)
   b. Somewhat representative of the average in the target population ★ (non-random sampling)
   c. Selected group of users / convenience sample
   d. No description of the sampling strategy
2. Sample size
   a. Justified and satisfactory ★
   b. Not justified / no information
3. Non-respondents
   a. Proportion of target sample recruited attains pre-specified target or basic summary of non-respondent characteristics in sampling frame recorded. ★
   b. Unsatisfactory recruitment rate; no summary data on non-respondents.
   c. No information provided
4. Ascertainment of the risk factor ('exposure')
   a. Validated screening tool used, or tool was validated in the study ★★
   b. Non-validated screening tool used, but the tool is available or described ★
   c. No description of the screening tool

Comparability

1. The participants in different outcome groups are comparable, based on the study design or analysis. Confounding factors are controlled for.
   a. The study controls for the most important factor ★
   b. The study controls for any additional factor ★

Outcome

1. Assessment of the outcome
   a. Independent blind assessment by experienced clinician ★★
   b. Unblinded assessment using objectively validated methods ★★
   c. Used non-standard or non-validated methods
   d. No description
2. Statistical test
   a. The statistical test used to analyse the data is clearly described, appropriate, and measures of association is presented, including confidence intervals and probability level (*p* value) ★
   b. The statistical test is not appropriate, not described or incomplete

We did not specify the most important confounding factor due to the heterogeneity of the studies reviewed; the primary factor was identified for each study individually.

ii. NEWCASTLE-OTTAWA QUALITY ASSESSMENT SCALE FOR COHORT STUDIES

Note*: A study can be awarded a maximum of one star for each numbered item within the Selection and Outcome categories. A maximum of two stars can be given for Comparability.*

Selection

1. <u>Representativeness of the exposed cohort</u>
   a. Truly representative of the average child in the community★
   b. Somewhat representative of the average child in the community ★
   c. Selected group of users
   d. No description of the derivation of the cohort
2. <u>Selection of the non-exposed cohort</u>
   a. Drawn from the same community as the exposed cohort ★
   b. Drawn from a different source
   c. No description of the derivation of the non-exposed cohort
3. <u>Ascertainment of exposure</u>
   a. Secure record ★
   b. Structured interview ★
   c. Written self-report
   d. No description
4. <u>Demonstration that outcome of interest was not present at start of study</u>
   a. Yes ★
   b. No

Comparability

1. <u>Comparability of cohorts on the basis of the design or analysis</u>
   a. Study controls for the most important confounding factor ★
   b. Study controls for any additional factor ★

Outcome

1. <u>Assessment of outcome</u>
   a. Independent blind assessment ★
   b. Record linkage ★
   c. Self-report
   d. No description
2. <u>Was follow-up long enough for outcomes to occur</u>
   a. Yes ★
   b. No
3. <u>Adequacy of follow-up of cohorts</u>
   a. Complete follow-up – all subjects accounted for ★
   b. Subjects lost to follow-up unlikely to introduce bias – small number lost - >70% follow-up. or description provided of those lost ★
   c. Follow-up rate < 70% and no description of those lost
   d. No statement

iii. NEWCASTLE-OTTAWA QUALITY ASSESSMENT SCALE FOR CASE-CONTOL STUDIES

Note*: A study can be awarded a maximum of one star for each numbered item within the Selection and Outcome categories. A maximum of two stars can be given for Comparability.*

Selection

1. Is the case definition adequate?
    a. Yes, with independent validation ★
    b. Yes, e.g. record linkage or self-report
    c. No description
2. Representativeness of the cases
    a. Consecutive or obviously representative series of cases ★
    b. Potential for selection biases or not stated
3. Selection of controls
    a. Community controls ★
    b. Hospital controls
    c. No description
4. Definition of controls
    a. No history of disease (endpoint)★
    b. No description of source

Comparability

1. Comparability of cases and controls on the basis of the design or analysis
    b. Study controls for the most important confounding factor ★
    c. Study controls for any additional factor ★

Exposure

1. Assessment of exposure
    a. Secure record ★
    b. Structured interview where blind to case/control status ★
    c. Interview not blinded to case/control status
    d. Written self-report or medical record only
    e. No description
2. Same methods of ascertainment for cases and controls
    a. Yes ★
    b. No
3. Non-response rate
    a. Same rate for both groups ★
    b. Non-respondents described
    c. Rate different and no designation

APPENDIX D

i. SUMMARY OF NEWCASTLE-OTTAWA SCALE QUALITY ASSESSMENT FOR CROSS-SECTIONAL STUDIES

Study author, date, country	SELECTION (maximum 5 stars)				COMPARABILITY (maximum 2 stars)	OUTCOME (maximum 3 stars)		RATING	QUALITY
	Represen-tativeness	Sample size	Similarity of non-respondents	Ascertain-ment of risk factor		Outcome assessment	Statistical test		
Kakooza-Mwesige et al., 2014, Uganda	★	★	☆	★☆	★★	★★	★	8/10	Good
Bitta et al., 2021, Kenya	★	★	☆	★☆	☆☆	★★	★	6/10	Satisfactory
Kariuki et al., 2020, Kenya	★	★	★	★★	☆☆	★★	★	8/10	Good
Kind et al., 2017, Kenya	★	★	★	★★	★★	★★	★	10/10	Very good
Arinda et al., 2020, Uganda	★	★	☆	★★	★★	☆☆	★	7/10	Good
Mazibuko & Chimbari, 2020, South Africa	☆	☆	☆	★☆	★★	☆☆	★	4/10	Unsatisfactory
Venter et al., 2015, South Africa	☆	☆	☆	★★	☆☆	★★	★	5/10	Satisfactory
Du Toit et al., 2021, South Africa	★	★	☆	★★	☆☆	★☆	★	6/10	Satisfactory
Knox et al., 2018, South Africa	★	★	☆	★★	★	★★	★	8/10	Good

Descriptions of quality ratings: 9-10 = Very Good; 7-8 = Good; 5-6 = Satisfactory; 0-4 = Unsatisfactory

ii. SUMMARY OF NEWCASTLE-OTTAWA SCALE QUALITY ASSESSMENT FOR COHORT- AND CASE-CONTROL STUDIES

Study author, date, country	Study design	SELECTION (maximum 4 stars)	COMPARABILITY (maximum 2 stars)	OUTCOME (maximum 3 stars)	RATING	QUALITY
Devandra et al., 2013, Malawi	Case-control	★ Adequate case definition<br>★ Representativeness of cases<br>★ Selection of controls<br>★ Definition of controls	★ HIV<br>★ 2 siblings from same household	★Ascertainment of exposure<br>★Same method for cases & controls<br>☆ Non-response rate reported	8/9	Good
Shuffrey et al., 2021, South Africa	Part of cohort study	☆Representativeness of exposed<br>★ Selection of non-exposed<br>☆ Ascertainment of exposure<br>★Demonstrates that outcome not present at start of study	★ sex<br>★ maternal factors	☆ Assessment of outcome<br>★ Follow-up period long enough<br>☆ Adequacy of follow-up of cohorts	5 /9	Fair
Brittain et al., 2022, South Africa	Part of cohort study	★Representativeness of exposed<br>★ Selection of non-exposed<br>☆ Ascertainment of exposure<br>★Demonstrates that outcome not present at start of study	★ ACEs<br>★ Maternal mental health	☆ Assessment of outcome<br>★ Follow-up period long enough<br>★ Adequacy of follow-up of cohorts	7/9	Good

Descriptions of quality ratings:

Good Quality: 3-4 stars in Selection; 1-2 stars in Comparability; 2-3 stars in Outcome/Exposure

Fair Quality: 2 stars in Selection; 1-2 stars in Comparability; 2-3 stars in Outcome/Exposure

Poor Quality: 0-1 star in Selection; 0 stars in Comparability; 0-1 star in Outcome/Exposure
